# Benefits of anterior chamber paracentesis in the management of glaucomatous emergencies

**Published:** 2014

**Authors:** M Cioboata, A Anghelie, C Chiotan, R Liora, R Serban, C Cornăcel

**Affiliations:** * Emergency Eye Hospital, Bucharest; ** Prof. Dr. Matei Bals” National Institute of Infectious Diseases, Bucharest; *** Clinical Emergency Ophthalmology Hospital, Bucharest

**Keywords:** alternative technique for IOP reduction, efficiency ACP

## Abstract

Anterior chamber paracentesis (ACP) is an alternative technique to reduce intraocular pressure (IOP) in patients with acute closed-angle glaucoma (ACAG) and requires controlled drainage of aqueous humor from the anterior chamber using a needle or an incision paracentesis with a knife.

**Purpose**. Evaluation of efficacy and safety of AC paracentesis in acute increases of intraocular pressure.

**Method**. This was an observational study done on a group of 24 patients with acute, unilateral increases of IOP> 50mmHg.

IOP was measured before, 10 minutes, 1 day, 7 days and 30 days after ACP using applanation tonometry.

**Results**. Intraocular pressure decreased from an average of 52.5 mmHg to 25.5 mmHg to 17.5 mmHg at 10 minutes and 7 days from the ACP.

Anterior chamber paracentesis combined with glaucoma medication led to the cessation of symptoms in all patients included in the lot and the resolution of corneal edema in 20 cases.

**Conclusion**. ACP is safe and effective in acute increases of IOP in the acute form of the primary angle closure glaucoma but remains an adjunct to conventional therapy drug.

**Abbreviations**: ACP = anterior chamber paracentesis, IOP = intraocular pressure, ACAG = acute closed-angle glaucoma

## Introduction

ACP is an alternative technique to reduce IOP in patients with ACAG and involves a controlled drainage of the aqueous humor from the anterior chamber by using a needle or a paracentesis incision. This can be done by using a slit lamp under topical anesthesia and aseptic conditions. ACP has the advantage of a rapid IOP reduction and repeatability of the procedure. Once IOP is reduced to acceptable levels (almost immediately), the grade of the corneal edema can allow a definitive treatment by laser Iridotomie which can be performed more quickly [**1**].

Literature does not offer too many details about the procedure. This may be due to the technical difficulties involved but also to the existence of the other methods of treatment already well documented. ACP should be performed with extreme caution due to technical difficulties related to the patient but also due to the ocular factors. The patient may have a marked discomfort with nausea and vomiting and may not cooperate, so some authors recommend performing the surgery under local anesthesia [**6**].

The anterior chamber can be very small, presenting a risk of infringement of the iris, lens or corneal endothelium. As a complication, malignant glaucoma may result from the rapid movement of the iris and lens towards [**3**-**5**].

Rapid decompression can lead to intraocular hemorrhages or more frequently decompression retinopathy and hyphaema [**5**].

Although rare, the last noteworthy complication is exogenous endophthalmitis [**3**].

Currently, this procedure has to address patients in whom the medical treatment is either unavailable or insufficient or the patients with the maximum level of medication without the control of the attack [**2**].

## Method

This was an observational study done on a group of 24 patients with acute, unilateral increases, IOP> 50mmHg, who presented to the emergency room of The Emergency Eye Hospital in Bucharest.

IOP was measured before, 10 minutes and 1 day, 7 days and 30 days after rACP by using the applanation tonometry.

Technique: The patient received acetazolamide (500mg orally) and lied down to be administered antibiotic and topical pilocarpine from 5 to 5 minutes. After this, he will sit at the slit lamp, slit positioned on AC and peripheral cornea.

Once sterilized with some Betadine, the surgeon would puncture the cornea with a 26 gauge needle at 9 o'clock for the right eye and 3 o’clock for the left one. 0.5 cc of aqueous humor will be aspirated until the iris came in front and contracted. A great care should be taken so as not to touch the lens. The topical antibiotic will be applied again from 5 to 5 minutes.

## Results

Patients were divided according to the clinical presentation in patients with ACAG (16 cases) and patients with acute secondary glaucoma (8 cases).

Intraocular pressure decreased from an average of 52.5 mmHg to 25.5 mmHg to 17.5 mmHg at 10 minutes and 7 days from the ACP.

Anterior chamber paracentesis combined with glaucoma medication led to the cessation of symptoms in all the patients included in the lot and the resolution of corneal edema in 20 cases.

There were four failures of ACP in patients with secondary glaucoma.

Of the 20 cases with corneal edema resolution, a total of 16 patients underwent Laser peripheral iridotomy.

We have not reported any complication related to ACP during the study.

## Conclusions

ACP is safe and effective in acute increases of IOP in acute primary angle closure glaucoma but remains an adjunct to conventional drug therapy. 

It still remains to be evaluated on a more important lot of patients.
